# Man or machine? Impact of tutor-guided versus simulator-guided short-time bronchoscopy training on students learning outcomes

**DOI:** 10.1186/s12909-021-02526-w

**Published:** 2021-02-22

**Authors:** Anke Schertel, Thomas Geiser, Wolf E. Hautz

**Affiliations:** 1 Department of Pulmonary Medicine, Inselspital, Bern University Hospital, University of Bern, Bern, Switzerland; 2 Department of Emergency Medicine, Inselspital, Bern University Hospital, University of Bern, Bern, Switzerland

**Keywords:** Bronchoscopy, Simulator training, Tutor, Supervision, Teaching method

## Abstract

**Background:**

Simulation based medical education is efficient for the acquisition of flexible bronchoscopy navigational skills and the knowledge of the tracheobronchial anatomy. However, bronchoscopy simulator training is not routinely integrated into pneumologic fellowship programs or undergraduate medical education for time and/or cost reasons. Our study compares the effect of self-guided bronchoscopy simulator training versus tutor guided training on the acquisition of navigational skills and knowledge of the bronchial anatomy.

**Methods:**

Third-year undergraduate medical students were randomized to either a tutor- or simulator guided bronchoscopy simulator training focusing on the acquisition of navigational skills and the knowledge of the tracheobronchial anatomy. Every student performed a baseline bronchoscopy followed by a structured bronchoscopy simulator training and finally an assessment bronchoscopy at the end of the training program. Groups were compared by means of a repeated measurement ANOVA and effect sizes calculated as Cohens’ *d.*

**Results:**

Fifty-four eligible students participated in the study. Knowledge of the tracheobronchial anatomy significantly increased from pre- to post training (all *p* < 0.001; all *d* > 2), navigational skills significantly decreased (all *p* < 0.005; all *d* < 1). There were no significant differences between groups. Instruction by the simulator as well as by the tutor was rated as helpful by the students. Twenty-two (84.6%) of the participants of the simulator guided group would have appreciated an additional instruction by a tutor.

**Conclusion:**

Short-time simulator guided bronchoscopy training improves knowledge of the tracheobronchial anatomy in novice bronchoscopists as much as tutor guided training, but navigational skills seem to worsen in both groups. Further studies assessing transfer to clinical practice are needed to find the optimal teaching method for basic flexible bronchoscopy.

**Supplementary Information:**

The online version contains supplementary material available at 10.1186/s12909-021-02526-w.

## Background

Flexible bronchoscopy (FB) is a safe procedure with low mortality and complication rates [1, 2], but the historical “in patient bronchoscopy education” poses a risk for both the learner and the patient. It can cause procedural anxiety, inappropriate diagnostic or therapeutic results, postprocedural complications and reduced learning opportunities in practices with low case loads [3].

Simulation based medical education is well established for the acquisition of procedural skills and superior to the traditional Halstedian “see one – do one – teach one” approach [4]. Simulation based bronchoscopy training improves patient outcomes [4] and procedure time [5]. With respect to educational outcomes, Gopal et al. found a significant improvement of anatomical knowledge and bronchoscopy navigation skills in medical students after a self-directed bronchoscopy simulator training (BST) [3]. Numerous studies showed, that novice bronchoscopists benefit from simulator training, but exact duration of training to achieve proficiency is unclear [3]. Ost et al. further found, that the skills acquired during BST transfer into real-patient bronchoscopy-performance [6]. Consequently, the CHEST Expert Panel Report on Adult Bronchoscopy Training suggests, that simulation should be part of a structured bronchoscopy teaching curriculum [7].

However, BST is not yet widely implemented in medical education. One of the barriers to its use is surely the cost of the currently available high-fidelity simulators, ranging between 20,000 to > 100,000 US$. Though, there is to mention that several trials have shown, that also low fidelity simulators, as 3D-printed models of the bronchial tree are effective for acquiring basic flexible bronchoscopy skills [8–11] by presenting an equal or even more realistic imaging compared to the high-fidelity models [9, 10, 12]. Other, less investigated models are available, for example one study used a low-cost device that simulates an intubated and ventilated patient, employing re-useable, inflatable, BioFlex-preserved, porcine lungs, with similar effects on trainee performance realism of the bronchial tree and usability [13]. For programs with budgetary constraints those models can be an alternative [14], as costs are significantly lower, ranging from 40 to 250 US$ [9].

Another barrier is, that, as physicians’ workload is generally high, it is difficult to provide time slots to integrate simulator training into existing curricula.

Physician supervisors however are commonly assumed to be required to provide simulation training, mostly because they provide expert feedback and because feedback is known to have a strong impact on learning [15].

However, the source of feedback seems to be less important than its presence [3]. Technically, feedback could come from different sources, e.g. from a supervisor, colleague or even the simulator itself [16]. Several trials have examined the effect of unsupervised self-guided BST [3, 5, 17], compared to no training [18]. For more complex surgical skills, additional mentoring seems to improve the learning outcome of robotic surgery simulation training, compared to self- guided training [19]. For FB to the best of our knowledge, no study has yet compared the effect of the presence of a supervisor to non-supervised simulator training on predefined learning outcomes. Our study thus aims to investigate whether simulator-guided bronchoscopy training can achieve similar acquisition of FB navigational skills and knowledge of the bronchial anatomy like tutor-guided training. If so, this could be a possibility to facilitate implementation of BST more widely and independent of supervisor availability.

## Methods

### Study type and objectives

This prospective randmomized controlled study compares two groups of novice bronchoscopists, that complete the same bronchosopy simulator training, one group guided by the simulator only, the other group with additional instruction by an experienced bronchoscopist as a tutor. The primary outcomes are the acquisition of FB navigational skills and knowledge of the tracheobronchial anatomy down to the level of bronchial segments.

### Setting

The study took place between November 25th and December 10th 2019 in the “Center of Bronchoscopy” of the Department of Pulmonary Medicine, University Hospital, Inselspital Bern, Switzerland on a portable high-fidelity bronchoscopy simulator (“3D Systems/Simbionix BronchExpress®”, 3D Systems – Simbionix, Airport City 70,151, Israel).

### Participants/recruitment

Third year undergraduate medical students of the University of Bern participating in a mandatory seminar on respiratory medicine were invited by E-Mail to participate in this study.

#### Ethics and consent to participate

Participation in the study was voluntary. Swiss research legislation (Human research ordinance HRO, §2) deems studies such as ours, which do not collect health related individual data, exempt from full ethical review. Consequently, this study has not been submitted for review to the Ethics Committee for Research of the Cantone of Bern). Nevertheless, at the beginning of each training session, participants were instructed about the objectives and purpose of the training and provided written informed consent**.**

### Study organization

#### Randomization and briefing

Upon arrival on the study site, students were randomized to either the tutor-guided group or the simulator-guided group by drawing a paper lot. Both groups received an introduction to the simulator and its operation by watching two short videos, followed by another 5-min in-person introduction of the simulator and the course of the simulator training. As the simulators’ program uses English abbreviations for the anatomical structures, a flipchart with explanations of the abbreviations was placed visibly in the training room, to reduce the cognitive load for our German-speaking students.

#### Pre-training assessment flexible bronchoscopy

Before beginning of the training, each student performed a 5-min bronchoscopy. Navigational skills (operationalized as percent of time in mid lumen; percent of time with scope-wall contact) and knowledge of the tracheobronchial anatomy (bronchial segments inspected/skipped; bronchial segments correctly identified) were recorded by the simulator.

#### Bronchoscopy simulator training

This assessment was followed by the training session. The simulator-guided group trained on the simulator by completing the curriculum given by the simulator. The tutor-guided group underwent the same curriculum but was supported by a tutor (AS), with a particular focus on navigational skills and orientation within the endobronchial system.

The training session consisted, first, of an exercise for basic scope manipulation in a non-anatomic environment. This was followed by a training within an anatomic environment, consisting of 5 consecutive 3-min-sessions, during which all anatomical structures were labeled by the simulator. Feedback was provided by the simulator in two ways. First, during the exercise for basic scope manipulation, students had to follow a ball with the scope in a metal tube system. The simulator played a metallic noise whenever the scope touched the wall. Second, by at the end of every session the simulator provided a table, listing the above mentioned outcome parameters (percent of time in mid lumen; percent of time with scope-wall contact, bronchial segments inspected/skipped, and bronchial segments correctly identified).

#### Post-training assessment

The training session was followed by a post-training-assessment, that was identical to the pre-training-assessment.

#### Questionnaire

Additionally, we developed a custom questionnaire assessing baseline characteristics, preexisting bronchoscopy experience, anatomical knowledge and feedback to the study session on 5 point Likert scales. A translated version of the original questionnaire is available as supplemental information.

### Data management and analysis

The simulator recorded all relevant performance data from pre-, and post-assessment as well as during training. Data were exported into an excel file and merged with student questionnaire data.

Student characteristics are reported as numbers, frequencies, percentages and mean or median and standard deviation or IQR, as appropriate.

Groups were compared by means of a repeated measurement ANOVA with type of training as the between subject factor and time of measurement (i.e. pre- or post-assessment) as within subject factor. A p-level of < 0.05 was set as significant. Effect sizes were calculated as Cohens’ *d*.

## Results

### Student characteristics

Out of 140 eligible students, 54 students participated. Their baseline characteristics are shown in Table 1.

**Table 1:** Baseline characteristics of participants

Participants	Tutor- Guided Group	Simulator-Guided Group
	*n* = 28	*n* = 26
**Age (years; median (IQR)**	22.8 IQR (21.0, 23.25)	22.1 (IQR 21.0, 23.0)
**Gender**		
**Male**	11 (39.3%)	8 (30.8%)
**Female**	17 (60.7%)	18 (69.2%)
**Right-handed**	24 (85.7%)	23 (88.5%)
**Left-handed**	4 (14.3%)	3 (11.5%)
**Bronchoscopy Experience**		
**No**	28 (100%)	24 (92.3%)
**Yes**	0	2 (7.7%)
**Self-assessed Knowledge of Tracheobronchial Anatomy**		
**None**	2 (7.1%)	2 (7.7%)
**Bad**	10 (35.7%)	10 (38.5%)
**Medium**	16 (57.1%)	14 (53.8%)
**Good**	0	0
**Very good**	0	0

### Navigational skills

There were no differences in navigational skills between groups before start of the training (Table 2). After training, both groups had significantly less time in mid-lumen in post-assessment compared to pre-assessment (*p* = 0.04) and significantly more scope wall contacts (*p* = < 0.001); effects were of medium (Cohens’ *d* = 0.52) and large size, respectively (*d* = 0.99). We did not observe a significant effect of type of training on acquisition of navigational skills (Table 2).

**Table 2:** Knowledge of endobronchial anatomy and navigational skills by time of measurement and group

	pre	post	***p-value***^**a**^	Cohens’ ***d***^**b**^
**Knowledge of endobronchial anatomy**				
**Segments correctly identified on 1st attempt (all)**	**4.38 (1.77)**	**12.04 (5.13)**	**< 0.001**	**2.21**
Simulator-guided	4.36 (1.66)	10.88 (4.83)	0.07	0.63
Tutor-guided	4.39 (1.81)	13.11 (5.26)		
**Segments correctly identified on any attempt (all)**	**5.58 (2.34)**	**13.83 (5.09)**	**< 0.001**	**2.15**
Simulator-guided	5.56 (2.29)	12.8 (5.06)	0.105	0.52
Tutor-guided	5.61 (2.42)	14.78 (5.06)		
**Segments skipped (all)**	**22.45 (2.31)**	**14.63 (4.72)**	**< 0.001**	**2.23**
Simulator-guided	22.48 (2.26)	15.48 (4.69)	0.14	0.46
Tutor-guided	22.43 (2.41)	13.85 (4.69)		
**Navigational skills**				
**% time in mid-lumen**	**44.04 (12.66)**	**37.63 (12.18)**	**0.004**	**0.52**
Simulator-guided	43.68 (12.52)	37.52 (16.37)	0.998	0.04
Tutor-guided	44.36 (13.02)	37.74 (13.85)		
**Scope wall contacts**	**15.36 (8.05)**	**23.63 (8.68)**	**< 0.001**	**0.99**
Simulator-guided	16.36 (7.54)	23.0 (9.04)	0.36	0.37
Tutor-guided	14.5 (8.53)	24.26 (8.47)		

^a^bold for comparison between pre- and post-assessment, normal for interaction between groups and time of measurement

^b^bold for comparison between pre- and post-assessment, normal for difference in gain/loss between groups

### Knowledge of tracheobronchial anatomy

**Fig. 1:**
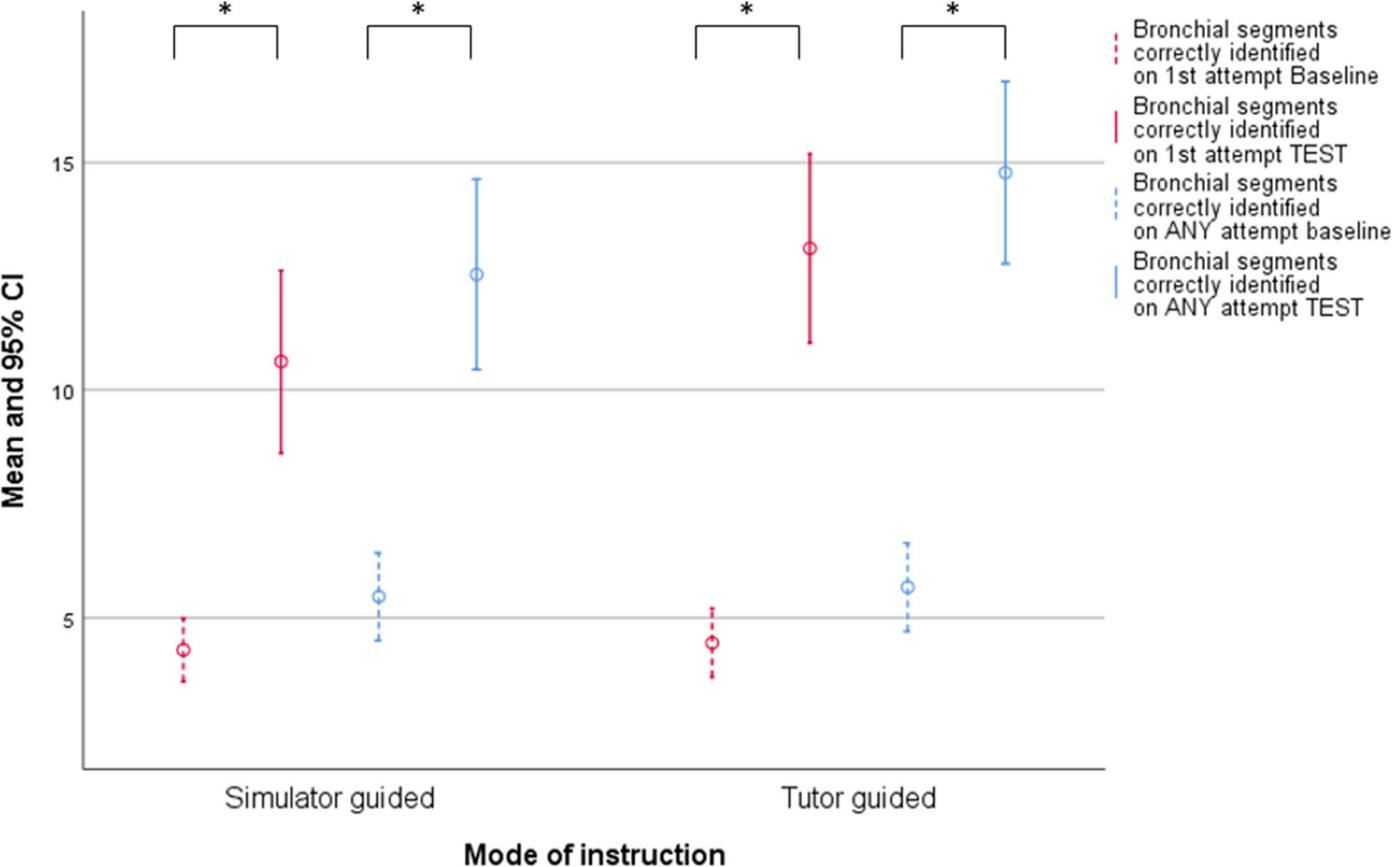
Effect of training on number of bronchial segments correctly identified by group. All significant differences marked *

There were no differences in knowledge of tracheobronchial anatomy between groups before start of the training (Table 2 and Fig. 1). After training, both groups correctly identified considerably more and skipped considerably less bronchial segments in post-assessment compared to pre-assessment (see Table 2 and Fig. 1; all *p* = < 0.001). This effect was very large (all *d* > 2). We did not observe a significant effect of type of training on acquisition of tracheobronchial anatomical knowledge (all *p* > 0.05; see Table 2), although the tutor-guided group performed somewhat better than the simulator-guided group on post-assessment (all *d* ~ 0.4; see Table 2).

### Questionnaire

All 28 participants in the tutor-guided group valued the instruction by the tutor as helpful (*n* = 17 (60.7%) or very helpful (11 (39.3%)) on a Likert scale.

In the simulator guided -group, the support by the simulator was rated as follows: very helpful: 4 (15.4%); helpful: 16 (61.5%); rather helpful: 4 (15.4%); a little helpful: 2 (7.7%), not helpful: 0 (0%). 22 (84.6%) of the participants in the simulator guided group would have liked an additional instruction by a tutor.

## Discussion

Our study found significant and large improvements in knowledge of the tracheobronchial anatomy after a short bronchoscopic simulator training. We did not observe any significant differences in this effect between modes of instructions, i.e. between simulator guided and tutor guided groups. Gains in anatomical performance were accompanied by significant and relevant drops in handling of the bronchoscope. Trainees spend less time in the mid lumen and had substantially more wall contacts. Again, this effect was independent of the mode of instruction.

There are at least two potential reasons why we did not find significant differences between modes of instruction. There either simply may be no relevant difference between the two groups, or our sample size may be too small to detect a difference of relevance. Indeed, the tutor guided groups shows larger gains between pre- and post-assessment than the simulator guided group, with medium effect sizes ranging between 0.46 and 0.63. The effects by the training per se however are at least 3.5fold larger than those of the mode of instruction. This, together with the fact that in clinical practice providing a tutor requires much more resources, may justify the use of simulator-guided instruction only.

The slightly larger gain in anatomical knowledge in the tutor-guided group comes at the expense of slightly worse navigational skills. Students in the tutor-guided group had slightly more scope wall contacts in post-assessment than those in the simulator-guided group (*d* = 0.37, a medium effect size). However, again, the effect of training per se is larger than the effect of the mode of instruction. In contrast to other studies [5, 17]_,_ that showed an improvement of navigational skills after a bronchoscopy simulator self-training, our results show the opposite. One possible reason is the short training time in our trial. Another aspect might be, that the students concentrated more on the identification of the bronchial segments to the disadvantage of the scope manipulation.

Training time in published bronchoscopy simulation studies range from a single 1-h-session to 10 repetitive training sessions [20]. For example, participants of an *8-h* simulator training session improved their navigational skills and missed fewer segments in the post-training assessment [17]. Because physician and student workload is generally high, it is difficult to provide time slots for regular simulator training within existing curricula. Our study could achieve improvement of anatomical knowledge and accuracy of tracheobronchial inspection after only a 1-h-simulator training, admittedly at the expense of navigational precision. An implication of our finding might be, that shorter training sessions should focus on only one or few objectives, e.g. either scope manipulation or identification of the anatomical structures.

Another point to discuss is the fact, that body posture, body language and movements, and the correct technique of advancing the scope without distortion cannot be observed by a simulator without a supervisor. Nevertheless these are important aspects of FB, that are frequently not intuitive for novice bronchoscopists and an accurate scope handling technique is mandatory for the performance of a high quality FB. Furthermore, 84.6% of our participants of the simulator-guided group would have appreciated the presence of an instructor for provision of guidance in handling of the scope. There may be a solution for that problem, as Collela et al. investigated a motion analysis system, that is able to provide automated feedback on correct movements during self-directed training on simulators. The authors commented that this approach opens the opportunity for trainees in bronchoscopy to receive automated feedback on their scope handling during training, without the need of an instructor being present [21].

Either way, feedback is frequently rated as one of the cornerstones of medical education [22], whereas the source of feedback seems to be less important [3]. In our study, the SIM group received feedback solely from the simulator, the TUT group from the simulator and the supervisor. Although not significant, but the TUT group had a slightly better performance in identifying the endobronchial segments. That is in line with Lee et al., who demonstrated that a trainee group with additional personal mentoring in robotic surgery training achieved significant better learning outcomes compared to self-directed, mentor-free learning [19]. This result is reflected in our finding, that 22 (84.6%) of the participants in the simulator guided group would have preferred an additional person-to-person mentoring.

However, this approach is again conflicting with limited availability of supervisors due to their workload and thus shortage of training time [19]. Being aware that additional supervision can enhance the learning experience and outcome, several studies, including ours, have shown a certain efficiency of self-directed simulator training compared to no training [23]. Thus, one can argue, that, in place of not offering simulator training due to lack of a tutor, self-directed training can be a feasible compromise.

Earlier trials have frequently used the Bronchoscopy Skills and Tasks Assessment Tool (BSTAT) to evaluate proficiency in FB [3, 24]. We intentionally decided to use the built-in assessment tool of the simulator, to again avoid the dependency of the presence of a human supervisor/assessor.

Our study has several limitations. First, the duration of the training session was short, with approximately 1 h. However, this conforms to reality where time for simulator training is limited. Second, our study consisted of only one single training session with immediate assessment afterwards and did not assess long-term retention. However, we primarily aimed to assess the presence and extent of a difference in learning between the tutor-guided and the simulator-guided group. Additionally, simulator training cannot completely replace clinical practice in real patients, where complications have to be managed, results have to be integrated and interpreted and patients have to be educated. All these tasks require training by a human instructor. When basic proficiency in FB is achieved through simulator training, parallel performance of “real bronchoscopy” will take place. This would interfere with any results of “long-term-skills-retainment-assessment of simulator training between the two groups. Furthermore it is known, that the learning curve is most effective in novices and then a certain plateau will be reached [5, 25, 26]. Thus, it can be assumed that in case of a difference between simulator and tutor guided simulation training, it would most likely be apparent in the early training stages.

Third, in real life, all bronchial segments have to be investigated and procedural performance is more likely to be reflected by the time needed for this procedure than by the number of segments skipped. However, we intentionally limited the time available for the examination to 5 min per assessment, so that the outcomes of interest could be measured on a single scale (i.e. number of segments identified or skipped) rather than having to adjust for two different scales (i.e. number of segments and time for the procedure). Earlier trials indeed found a reduction in procedure time after BST [5]. A shorter procedure time may result in better patient comfort [27], and less complications [5, 28]. However, despite the advantages of a short procedure time, we argue that the patient benefits even more from a cautious and atraumatic scope manipulation and a precise and complete inspection of the bronchial system.

Last, bronchoscopy skills may be more relevant for postgraduates than for students, especially for respiratory physicians. However, most of the residents in respiratory medicine have never previously learned bronchoscopy. Thus, we took undergraduate students as a surrogate for novice bronchoscopists, who, according to the available data, benefit most from bronchoscopy simulator training, compared to more experienced physicians.

Despite these limitations and the necessity of further research, in times of high physician workload, scarcity of time, money and availability of educators, our study is an important step on the way to determine the ideal teaching approach for basic FB.

## Conclusion

Short time BST is effective in improving the performance of FB tracheobronchial inspection and can thereby enhance the knowledge of the tracheobronchial anatomy. Although the majority of participants of the simulator-guided would have appreciated an additional instruction by a human person, this effect is largely independent of the presence of a human supervisor. Instead, feedback, the most powerful tool for enhancement of learning, is provided by the simulator. Our findings justify the implementation of short-time (1 h) self-guided BST into medical education, whereas shorter training sessions should focus on only one or few learning objectives. More empirical studies are needed to determine, if sparing a supervisor and thereby human resources may facilitate the implementation of BST into existing curricula. Further studies are necessary to determine the optimal teaching modalities for the acquisition of basic FB skills.

## Supplementary Information

**Additional file 1.**

**Additional file 2.**

## Data Availability

The datasets used and/or analyzed during the current study are available from the corresponding author on reasonable request.

## Abbreviations

BST: Bronchoscopy Simulator Training
FB: Flexible Bronchoscopy
