# Supplementary material for: Man or machine? Impact of tutor-guided versus simulator-guided short-time bronchoscopy training on students learning outcomes
Source: BMC Med Educ. 2021 Feb 22;21:123. doi: 10.1186/s12909-021-02526-w (PMC7898762; doi:10.1186/s12909-021-02526-w)
Supplement: Supplementary file 1 — Additional file 1. [file 12909_2021_2526_MOESM1_ESM.docx]

# **Questionnaire Without Tutor**

A

**Participant**

**Number:**

**Participant**

**Number:**

# **Self-Guided vs. Tutor-Guides Simulator Training for Basic Bronchoscopy**

Thank you for completing the following questionnaire.

| **Surname:** |  | **Name:** |  | **Age:** |  |
| --- | --- | --- | --- | --- | --- |

**Gender:**  female  male

**Handedness:**  righthanded  lefthanded

**Smoking:**  yes  no

**Year of Study:**  1  2  3  4  5  6

1. **How often do you play video games in your leisure time? (e.g. Playstation, X-Box )?**

never rarely (ca. 1x / month) sometimes (ca. 1x/ month) often (ca. 1x/ week) very often (>1x/week)

〇--------------------〇------------------〇----------------〇--------------〇

1. **Have you already decided for a subspecialty in medicine after you have finished your studies?**

ja  nein

1. **If yes, for which one?**

|  |
| --- |

1. **How intense is your interest in performing medical interventions (such as bronchoscopy, angiography, gastroscopy, laparoscopy) in your future professional life?**

none low medium high very high

〇-------------〇-------------〇-------------〇-------------〇

1. **Have you ever done a bronchoscopy before ?**

yes  no

1. **How do you rate your current knowledge of the tracheobronchial anatomy?** (Please fill in here the result of the question on the paper sheet, you answered before the training)

none bad medium good very good

〇-------------〇-------------〇-------------〇-------------〇

1. **How do you rate your knowledge of the tracheobronchial anatomy now after the simulator training?**

none bad medium good very good

〇-------------〇-------------〇-------------〇-------------〇

1. **How much fun did you experience during the simulator training?**

No fun little medium much very much

〇-------------〇-------------〇-------------〇-------------〇

1. **How helpful do you rate the support by the simulator?**

Not helpful little medium helpful very helpful

〇-------------〇-------------〇-------------〇-------------〇

1. **Would you have wished to have additional support by a supervisor/tutor?**

yes  no  I don’t know

**If yes, why?**

**Thank you very much for your participation!**
